# DRP1-Mediated Mitochondrial Fission Regulates Lung Epithelial Response to Allergen

**DOI:** 10.3390/ijms222011125

**Published:** 2021-10-15

**Authors:** Sierra R. Bruno, Amit Kumar, Zoe F. Mark, Ravishankar Chandrasekaran, Emily Nakada, Nicolas Chamberlain, Bethany Mihavics, Joseph Walzer, Jonathon Cahoon, Anne E. Dixon, Brian Cunniff, Vikas Anathy

**Affiliations:** 1 Department of Pathology and Laboratory Medicine, Larner College of Medicine, University of Vermont, Burlington, VT 05405, USA; srbruno@uvm.edu (S.R.B.); amit.kumar@med.uvm.edu (A.K.); zoe.mark@med.uvm.edu (Z.F.M.); emily.nakada@mail.mcgill.ca (E.N.); nicolas.chamberlain@yale.edu (N.C.); bethany.korwin@uvm.edu (B.M.); joseph.walzer@uvm.edu (J.W.); jonathon.cahoon@cuanschutz.edu (J.C.); brian.cunniff@uvm.edu (B.C.); 2 Department of Medicine, Larner College of Medicine, University of Vermont, Burlington, VT 05405, USA; ravishankar.chandrasekaran@med.uvm.edu (R.C.); anne.dixon@uvmhealth.org (A.E.D.)

**Keywords:** mitochondrial fission, epithelial cell, HDM, allergic airway disease, DRP1

## Abstract

Mitochondria regulate a myriad of cellular functions. Dysregulation of mitochondrial control within airway epithelial cells has been implicated in the pro-inflammatory response to allergens in asthma patients. Because of their multifaceted nature, mitochondrial structure must be tightly regulated through fission and fusion. Dynamin Related Protein 1 (DRP1) is a key driver of mitochondrial fission. During allergic asthma, airway epithelial mitochondria appear smaller and structurally altered. The role of DRP1-mediated mitochondrial fission, however, has not been fully elucidated in epithelial response to allergens. We used a Human Bronchial Epithelial Cell line (HBECs), primary Mouse Tracheal Epithelial Cells (MTECs), and conditional DRP1 ablation in lung epithelial cells to investigate the impact of mitochondrial fission on the pro-inflammatory response to house dust mite (HDM) in vitro and in vivo. Our data suggest that, following HDM challenge, mitochondrial fission is rapidly upregulated in airway epithelial cells and precedes production of pro-inflammatory cytokines and chemokines. Further, deletion of *Drp1* in lung epithelial cells leads to decreased fission and enhanced pro-inflammatory signaling in response to HDM in vitro, as well as enhanced airway hyper-responsiveness (AHR), inflammation, differential mucin transcription, and epithelial cell death in vivo. Mitochondrial fission, therefore, regulates the lung epithelial pro-inflammatory response to HDM.

## 1. Introduction

Asthma is an inflammatory respiratory disease that is estimated to affect over 339 million people globally [1]. In roughly 50–60% of asthma patients, complex allergens such as house dust mite (HDM) exacerbate symptoms [2]. Epithelial cell mitochondria play a key role in the pathogenesis and progression of asthma and the allergic airway response [3,4,5,6]. It has long been documented that lung epithelial cells of asthmatics and mouse models of asthma have altered mitochondria with damaged cristae [7,8]. Recent studies have demonstrated that allergen-induced alterations in mitochondrial function drives epithelial pro-inflammatory signaling, apoptosis, and enhanced airway hyper-responsiveness (AHR) [3,8,9,10].

Structural changes to mitochondria that influence function, i.e., fission and fusion, are integral for mammalian cellular homeostasis and survival [11,12,13,14,15,16,17]. One of the key proteins necessary for mitochondrial fission is the GTPase Dynamin Related Protein 1 (DRP1). DRP1 is a cytosolic protein that, upon activation via phosphorylation and/or other post-translational modifications at key residues, localizes to the mitochondrial outer membrane at sites where the endoplasmic reticulum interacts with and preconstricts the mitochondrion. There, it interacts with different outer mitochondrial membrane-bound proteins, including FIS1 (mitochondrial fission protein 1), MFF (mitochondrial fission factor), MiD49 (mitochondrial dynamics protein 49) and MiD51 (mitochondrial dynamics protein 51), to organize into multimeric rings around the mitochondria and constrict the outer mitochondrial membrane in order to allow the separation of mitochondria into two segments (fission) [18,19,20,21,22]. Several studies have suggested that mitochondria appear visually smaller in the airway epithelia of asthmatics or asthma mouse models, as seen via transmission electron microscopy [7,8]; however, the role of epithelial DRP1-mediated mitochondrial fission has yet to be fully elucidated in the allergic airway disease.

This study aimed to determine whether exposure to a complex allergen induces mitochondrial fission, and to investigate the contribution of DRP1 mediated mitochondrial fission in the epithelial response to that allergen. We characterized the mitochondrial fission dynamics in airway epithelia after allergen exposure and elucidated potential mechanisms of action in the regulation of the allergic response initiated by epithelial cells.

## 2. Results

### 2.1. Human and Mouse Airway Epithelial Cells Upregulate DRP1-Mediated Mitochondrial Fission in Response to Allergen

**Figure 1:**
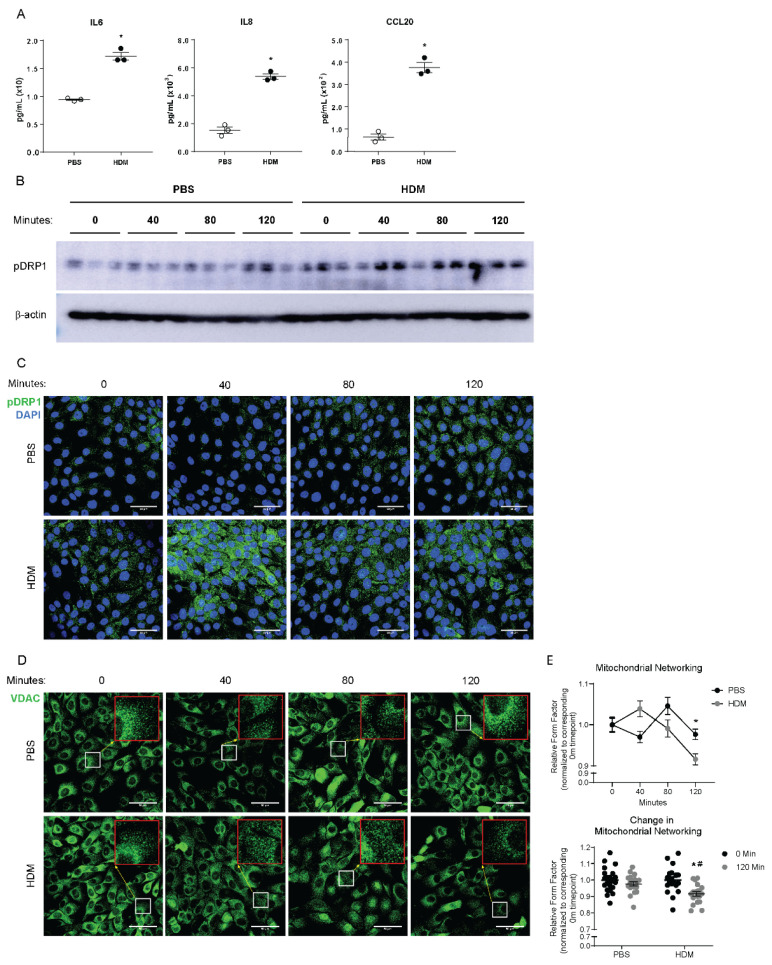
HDM induces mitochondrial fission in human bronchial epithelial cells. (**A**) ELISAs on supernatants from cultured HBECs, *n* = 3 per group; (**B**) Western blot analysis of pDRP1 in whole cell lysates, *n* = 3 per group; (**C**) Representative images of immunofluorescence of pDRP1 in fixed HBECs; (**D**) Representative images from immunofluorescence of VDAC in fixed HBECs with insets at 3× magnification in red; (**E**) Quantification of mitochondrial networking based on VDAC mitochondrial immunofluorescence, form factor calculation with *n* = 19 images per group at 60× magnification; (**A**,**B**,**E**) * *p* < 0.05 vs. PBS group, (**C**) * *p* < 0.05 vs. corresponding 0 Min group, # *p* < 0.05 vs. PBS 120 Min group. Error bars represent mean ± SEM. (**C**,**D**) Scale bars are 50 µm.

Previous literature suggests that in people with asthma, epithelial mitochondria have altered structure, including smaller overall size [7,8]. Thus, we aimed to determine whether DRP1-mediated mitochondrial fission is induced upon airway epithelial stimulation with HDM. We retrospectively analyzed a microarray dataset (GSE43696) retrieved through the NCBI Gene Expression Omnibus (GEO) to obtain a basal transcription of *DRP1* in the bronchial epithelial cells of human patients with moderate and severe asthma, as described by the American Thoracic Society [23,24]. The expression of two transcript variants of *DRP1*, both expressed in most human cell types, is modestly but significantly upregulated in severe asthmatic bronchial epithelial cells compared to non-asthmatic controls (Figure S1), indicating a potential role for DRP1 in regulation of asthma progression. The clinical significance of this is not yet clear. We believe additional retrospective analyses along with patient characteristics data are needed to correlate with the clinical significance. To further analyze the role of this upregulation, we assessed the activity of DRP1 in vitro in human bronchial epithelial cells (HBEs) exposed to HDM. HDM induces the secretion of pro-inflammatory cytokines and chemokines upregulated and released by epithelial cells 24 h after allergen exposure (Figure 1A). Phosphorylation of DRP1 at serine 616 (S616) has been shown to enhance GTPase activity of the protein [11,25,26]. We found that HDM exposure of HBEs acutely increased the phosphorylation of DRP1 at S616 at 40 min post-HDM exposure, and this phosphorylation dissipated within 120 min from HDM exposure (Figure 1B,C). Mitochondrial fission initiated by activated DRP1 can be determined by measuring average mitochondrial size [27,28]. This can also be quantified by form factor, a measurement of length and branching of the mitochondrial network [29,30,31]. Activation of DRP1 immediately precedes an induction of mitochondrial fission in HBEs exposed to HDM, as indicated by decreased form factor and average mitochondrial area at 120 min postexposure (Figure 1D,E). Next, to understand the role of mitochondrial fission in the pro-inflammatory response initiated by airway epithelial cells, we aimed to confirm that these processes also occurred in primary mouse tracheal epithelial cells (MTECs). Upon stimulation with HDM, MTECs respond similarly to human airway epithelial cells. Phosphorylation of DRP1 is increased 40 min after HDM exposure, and dissipates after 120 min (Figure S2A,B). As in human airway epithelial cells, activation of DRP1 precedes the pro-inflammatory response initiated by allergen stimulation (Figure S2C).

HDM is a complex allergen that includes components of bacterial, fungal, and HDM origins. One of the components of bacterial origin is lipopolysaccharide (LPS). LPS is known to induce DRP1 activation [32,33,34]. Therefore, we treated HBECs with LPS (0.6 ng/mL) at a level comparable to that found in the HDM used in our studies, as well as at a higher dose (100 ng/mL), to examine whether LPS alone is responsible for the DPR1 response induced by HDM. Phosphorylation of DRP1 at S616 does not appear to be increased by LPS at either concentration (Figure S3).

### 2.2. In Vitro Deletion of DRP1 Enhances the Pro-Inflammatory Response to Allergen

**Figure 2:**
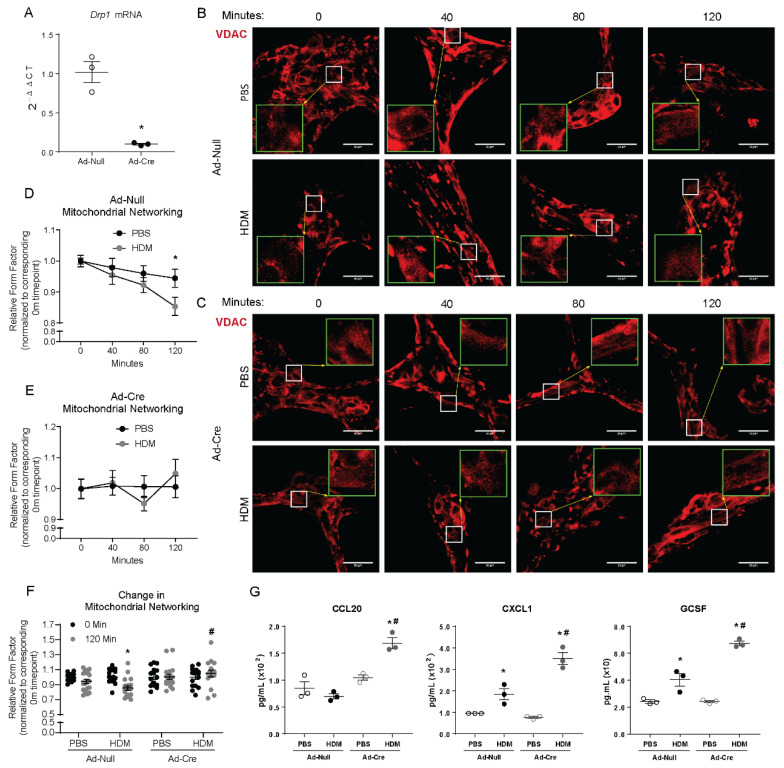
*Drp1* deletion impairs mitochondrial fission and enhances the mouse epithelial pro-inflammatory response to HDM. (**A**) RT-qPCR of *Drp1* following Cre-induced deletion in cultured MTECs, *n* = 3 per group; (**B**) Representative images of immunofluorescence of VDAC in fixed MTECs treated with Ad-Null with insets at 3× magnification in green; (**C**) Representative images of immunofluorescence of VDAC in fixed MTECs treated with Ad-Cre with insets at 3× magnification in green; (**D**) Quantification of mitochondrial networking based on VDAC immunofluorescence, form factor calculation with Null *n* = 19–21 images per group; (**E**) Quantification of mitochondrial networking based on VDAC immunofluorescence, form factor calculation with Cre *n* = 14–18 images per group at 60× magnification; (**F**) Overall change in mitochondrial networking, each data point represents 1 image; (**G**) ELISAs on supernatants from MTECs, *n* = 3 per group; (**A**) * *p* < 0.05 vs. Ad-Null group; (**B**,**C**) Scale bars are 50 µm. (**D**,**E**) * *p* < 0.05 PBS vs. HDM; (**F**) * *p* < 0.05 vs. corresponding 0 Min group, # *p* < 0.05 vs. Ad-Null HDM 120 Min group; (**G**) * *p* < 0.05 vs. corresponding PBS group, # *p* < 0.05 vs. Ad-null HDM group. Error bars represent mean ± SEM.

Considering DRP1-mediated mitochondrial fission occurs prior to the pro-inflammatory response initiated in airway epithelial cells, we sought to determine the role of fission in pro-inflammatory signaling. We utilized a gene deletion system whereby insertion of two Loxp sites allowed for cre recombinase-induced excision of a segment of DNA within the gene of interest. We determined that infecting MTECs containing *Drp1*^loxp/loxp^ sites with an adenovirus vector expressing cre recombinase (Ad-Cre) at an MOI of 8 significantly decreased *Drp1* mRNA levels to ~10% of adenoviral control (Ad-Null) levels (Figure 2A). While HDM induced mitochondrial fission in control cells, stimulation with HDM of *Drp1*-deleted MTECs did not induce a decrease in mitochondrial networking as determined by form factor, thus exhibiting an impairment in mitochondrial fission following *Drp1* deletion (Figure 2B–F). Using this system, we also analyzed the pro-inflammatory response of MTECs after deletion of *Drp1*. *Drp1*-deleted MTECs exposed to HDM exhibited a significant increase in the secretion of pro-inflammatory cytokines and chemokines 24 h postexposure to HDM (Figure 2G).

### 2.3. Conditional Deletion of Drp1 In Vivo Increases Inflammation in Response to Allergen Exposure in a Mouse Model of Allergic Airways Disease

**Figure 3:**
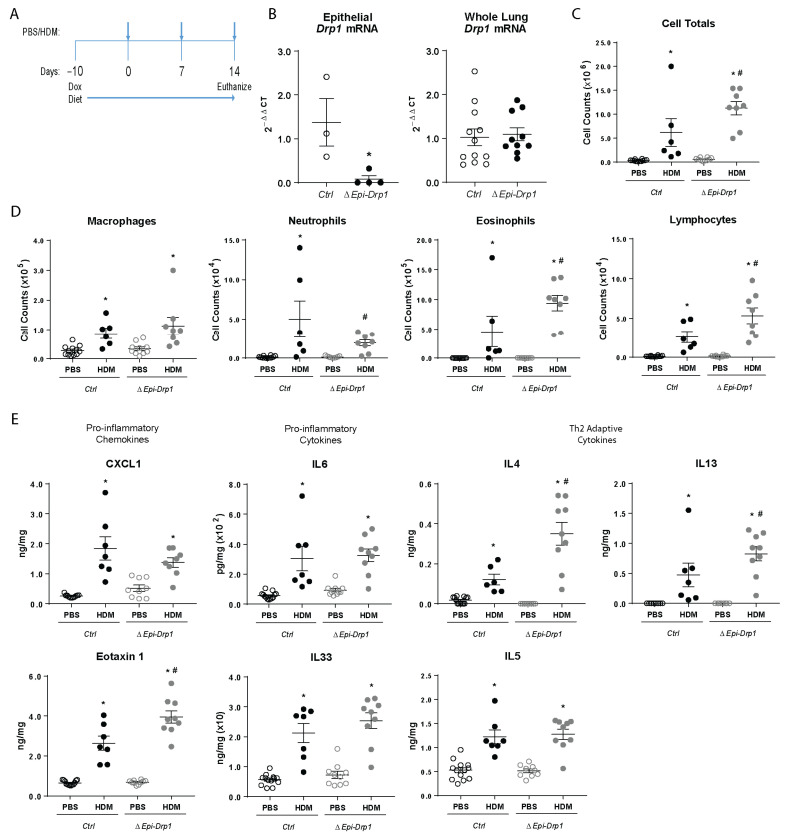
Epithelial specific *Drp1* deletion enhances inflammatory cell migration to mouse lungs in response to HDM. (**A**) Deletion and HDM exposure protocol; (**B**) RT-qPCR assessment of *Drp1* in lung epithelial lysates, *n* = 3 mice per group, and whole lung lysates, *n* = 10–12 mice per group from 2 experiments; (**C**) Total inflammatory cells present in the BALF, *n* = 6–12 mice per group from 2 experiments; (**D**) Inflammatory cell-specific totals in the BALF, *n* = 6–12 mice per group from 2 experiments; (**E**) ELISAs of inflammatory cytokines and chemokines in whole lung lysates, *n* = 6–13 mice per group from 2 experiments; (**B**–**E**) * *p* < 0.05 vs. corresponding PBS group, # *p* < 0.05 vs. *Ctrl* HDM group. Error bars represent mean ± SEM.

To understand the impact of the enhancement of the epithelial pro-inflammatory response to HDM due to the deletion of *Drp1*, we generated a CC10-rTetA/TetOP-Cre/*Drp1^loxp/loxp^* mouse to delete *Drp1* in lung epithelial (club) cells in the presence of doxycycline. Mice were put on a diet containing doxycycline 10 days before initiation of the allergic airways disease experimental protocol; the diet was continued until the end of the sensitization period and the challenge protocol. Mice were sensitized on days 0 and 7, followed by a single challenge on day 14 with 25 µg HDM, or phosphate buffered saline (PBS) control, intranasally. Mice were sacrificed 4 h after the single challenge to assess the role of DRP1 in initiation of the allergic airway response (Figure 3A). Isolated lung epithelial cells revealed significant reduction of *Drp1* transcript in the *ΔEpi-Drp1* mice compared to littermate control (*Ctrl*) mice. This reduction could not be seen, however, in whole lung lysate, confirming lung epithelial specific *Drp1* deletion (Figure 3B). The bronchoalveolar lavage fluid (BALF) was analyzed for immune cell infiltration into the lungs. This analysis revealed a significant increase in the total cell infiltrates to the lungs after HDM challenge with *Drp1* deletion compared to littermate controls (Figure 3C). Assessment of the cell types in the airways revealed eosinophils and lymphocytes were significantly upregulated in the lungs of *Drp1*-deleted mice challenged with HDM (Figure 3D). Whole lung tissue lysates were analyzed for pro-inflammatory cytokines, chemokines and Th2 cytokines via enzyme-linked immunosorbent assay (ELISA). There were no differences seen in production of several epithelial-secreted pro-inflammatory cytokines such as IL6 and IL33 or Th2 cytokine IL5 between *ΔEpi-Drp1* mice challenged with HDM, compared to HDM-challenged *Ctrls*. Alternatively, *ΔEpi-Drp1* mice challenged with HDM expressed higher levels of the pro-inflammatory eosinophil chemokine Eotaxin, and the Th2 cytokines IL4 and IL13 (Figure 3E). This could suggest a role for mitochondrial fission in regulation of specific epithelial pro-inflammatory signaling.

### 2.4. In Vivo Conditional Deletion of Drp1 Enhances Mucin Expression and Epithelial Apoptosis

**Figure 4:**
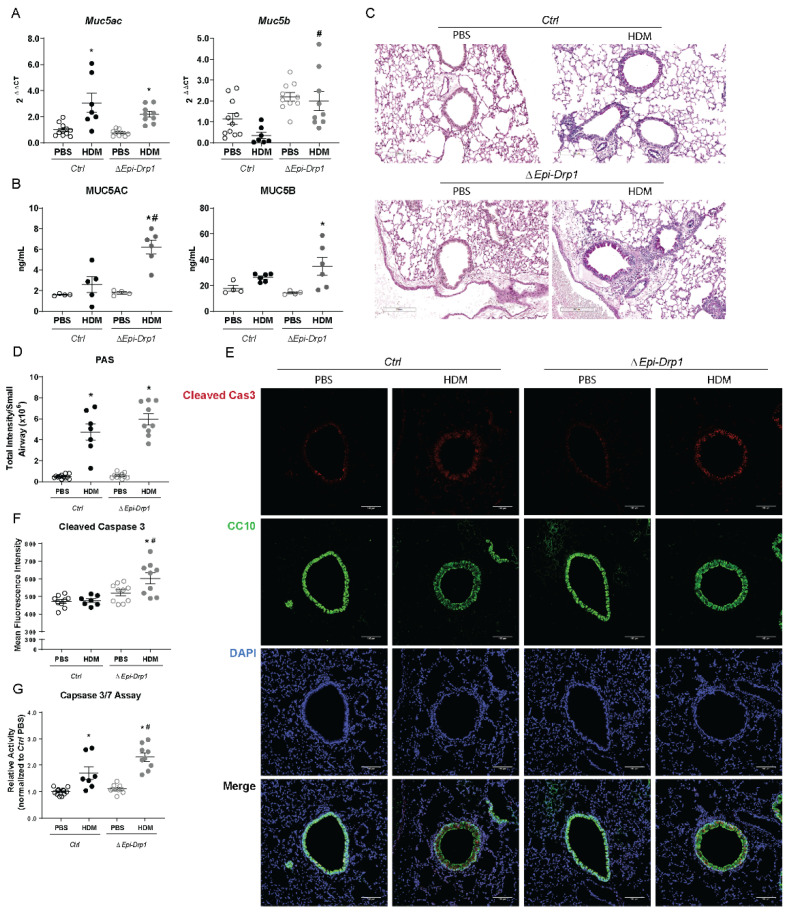
Epithelial specific *Drp1* deletion enhances mucin metaplasia and epithelial cell death following HDM exposure. (**A**) RT-qPCR of mucins in whole lung lysates, *n* = 6–12 mice per group from 2 experiments; (**B**) ELISAs of mucins in the BALF, *n* = 6–12 mice per group from 2 experiments; (**C**) Representative images of PAS staining in lung tissue sections; (**D**) Quantification of PAS staining, *n* = 7–12 mice per group from 2 experiments; (**E**) Representative immunofluorescence images of Cleaved Caspase 3 (Cleaved Cas3) and CC10; (**F**) Quantification of mean fluorescence intensity (MFI) of Cleaved Cas3 in immunofluorescence images, *n* = 7–10 mice per group from 2 experiments; (**G**) Luminescence activity assay of Caspases 3 and 7 (Caspase Glo Assay) in whole lung lysates, *n* = 7–13 mice per group from 2 experiments; (**A**,**B**,**D**,**F**,**G**) * *p* < 0.05 vs. corresponding PBS group, # *p* < 0.05 vs. *Ctrl* HDM group. Error bars represent mean ± SEM. (**C**) Scale bars are 200 µm. (**E**) Scale bars are 100 µm.

A common hallmark of allergen-induced asthma and an indicator of asthma severity is mucus metaplasia [35,36,37]. Therefore, we examined mucin expression in the lungs after *Drp1* deletion. While *Muc5ac* mRNA levels were not expressed at a higher level in *∆Epi-Drp1* mice compared to *Ctrl* HDM exposed mice, *Muc5b*, more commonly upregulated in fibrotic lung diseases and linked with severity of fibrosis [38,39,40,41], was significantly increased in both PBS and HDM groups compared to corresponding *Ctrl* groups (Figure 4A). We also examined mucin secretion into the BALF via ELISA. MUC5AC secretion was significantly enhanced in the HDM-exposed *ΔEpi-Drp1* mice compared the HDM-exposed *Ctrl* mice. MUC5B secretion, though not significant, trended higher in the *ΔEpi-Drp1* mice compared to *Ctrls* (Figure 4B). Also, mucus staining trended higher but was not significantly increased in the airways of *∆Epi-Drp1* mice compared to littermate controls, as demonstrated by Periodic Acid Schiff (PAS) (Figure 4C,D). These data suggest that epithelial DRP1 may regulate differential mucin expression, further suppressing severity of the allergic response to HDM.

Mitochondrial fission is believed to play a key role in cell death regulation [42,43,44]; therefore, we examined activation of the apoptosis regulator, Caspase-3, in mouse lung tissue. Immunofluorescence for active (cleaved) caspase-3 was conducted and analyzed, revealing an increased level of cleaved caspase-3 in the airway epithelia of *ΔEpi-Drp1* mice compared to littermate controls challenged with HDM, as indicated by increased relative mean fluorescence intensity (Figure 4E,F). Further, we performed a Caspase-Glo assay in mouse lung tissue and observed a significant increase in the activity of caspases in tissue lysates of *ΔEpi-Drp1* mice compared to *Ctrls* (Figure 4G), indicating increased apoptosis. These data together indicate a worsened allergic airway response to HDM after *Drp1* deletion with enhanced epithelial cell death and altered mucin profile.

### 2.5. Drp1 Deletion In Vivo Enhances Airway Hyperresponsiveness to Methacholine

**Figure 5:**
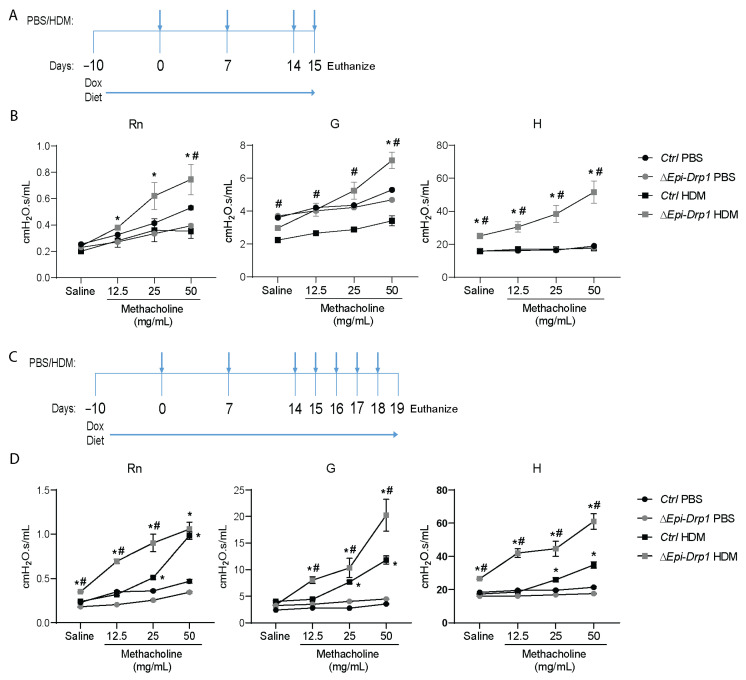
Epithelial specific *Drp1* deletion enhances AHR in mouse lungs in response to HDM. (**A**) Deletion and HDM exposure protocol for AHR initiation experiment; (**B**) Results from AHR initiation experiment, *n* = 5 mice per group from 2 experiments; (**C**) Deletion and HDM exposure protocol for established AHR experiment; (**D**) Results from AHR initiation experiment, *n* = 4 mice per *ΔEpi-Drp1* group and *n* = 6 for remaining groups from 2 experiments. (**B**,**D**) * *p* < 0.05 vs. corresponding PBS group, # *p* < 0.05 vs. *Ctrl* HDM group. Error bars represent mean ± SEM.

Next, we assessed methacholine-induced airway hyperreactivity (AHR) in the HDM-induced allergic airway disease model. Experiments were carried out with two challenges to allow more time to develop reactivity in the lungs (Figure 5A). While *Ctrl* mice sensitized and challenged with HDM did not have increased AHR (i.e., Rn, G, and H) as compared to PBS sensitized and challenged mice, *ΔEpi-Drp1* mice had significantly increased AHR in response to HDM (Figure 5B). AHR was also examined in a more established model of HDM inflammation to examine whether *Drp1* deletion also maintained enhanced HDM-induced AHR when compared to control mice that had also developed airway hyperresponsiveness (Figure 5C). Following this protocol, *Ctrl* HDM-challenged mice developed enhanced AHR compared to their PBS counterparts. In agreement with the previous experiment, *ΔEpi-Drp1* HDM-challenged mice had enhanced AHR compared to their PBS counterparts, and significantly increased AHR compared to *Ctrl* HDM-challenged mice (Figure 5D). These data indicate that increased inflammation and mucosal secretions induce a collapse in the peripheral airways, as seen in other models of acute allergen-induced inflammation [45]. These data also suggest a more severe response to allergen following *Drp1* deletion.

## 3. Discussion

We found that both human and mouse airway epithelial cells rapidly upregulate the phosphorylation of DRP1 at Serine 616 (S616), indicating a role in early response to the allergen. *Drp1* deletion in vitro and in vivo hindered mitochondrial fission and enhanced secretion of pro-inflammatory markers from epithelial cells following HDM exposure, as well as the activation of pro-apoptotic markers. *Drp1* epithelial deletion also increased AHR in mice, an indicator of airway remodeling during response to allergen insult. Finally, Muc5B transcription and apoptosis were also increased following *Drp1* epithelial deletion in HDM-challenged mice. Together, these data demonstrate a role for DRP1-dependent mitochondrial fission in the regulation and response of airway inflammation initiated by airway epithelia in response to complex allergen. Increases in DRP1 expression and activity, as well as mitochondrial structural alterations, have already been linked to airway inflammatory responses to various insults in multiple cell types [34,46,47,48,49]. The role that DRP1 plays in the initiation of epithelial response to HDM, however, had not been previously demonstrated. Therefore, herein we present data evaluating the role of DRP1, and mitochondrial fission, in the initiation of the airway allergic response to the complex allergen HDM in both in vitro and in vivo model systems.

Several studies have linked DRP1-mediated mitochondrial fission to activation of NF-κB, thus linking increased DRP1 expression with an enhanced inflammatory response to various stimuli in multiple cell types [50,51,52]. There is, however, a lack in understanding of the role DRP1 plays in inflammation initiated by airway epithelial cells in response to allergen. Though several studies have suggested DRP1 induces inflammatory signaling, here we show a link between DRP1 and suppression of HDM-induced pro-inflammatory cytokine and chemokine release by airway epithelia in culture. In agreement with our work, another study demonstrated DRP1 suppresses pro-inflammatory signaling in macrophages stimulated with LPS via regulation of release of mitochondrial damage-associated molecular patterns (DAMPs) [33]. Mitochondrial DAMPs play an important role in inflammatory signaling in response to lung injury [53,54,55]. Mitochondrial DAMP release could be suppressed by DRP1-induced fission in airway epithelia following HDM exposure, though more detailed studies are needed to examine this phenomenon. This scenario represents a signal-dependent dichotomous action of DRP1 in regulation of inflammatory signaling.

LPS has been shown to upregulate DRP1 activation and mitochondrial fission in various lung cell types [32,33,34]. Although HDM contains LPS, our data suggest that HDM-induced DRP1 activation is not entirely reliant on its LPS content. Previous studies examining LPS activation of DRP1 and induction of mitochondrial fission were done in different cell types using higher concentrations of LPS. While LPS may be an important signaling molecule in the HDM response, the LPS alone does not induce early activation of DRP1 at the concentrations present in HDM.

Mucus metaplasia can contribute to enhanced AHR [35,36,37]. Mucus is secreted by goblet cells, a specific subset of epithelial in the airways [56]. Our data suggest that the club cell specific deletion of *Drp1* increases mucin transcription and translation after HDM exposure. Club cells are known to act as progenitor-type cells, and can differentiate into numerous epithelial subtypes, including goblet cells [57]. Mitochondrial fission is also known to play a role in regulation of differentiation of various cell types [58,59,60]. These data suggest a link between mitochondrial fission and differentiation of club cells into pathological epithelial subtypes.

DRP1-mediated mitochondrial fission plays regulates programmed cell death as well as cell survival in stress environments [29,44,61,62,63,64,65,66]. DRP1 expression and mitochondrial fission have been linked to mitophagy of mitochondria damaged by oxidative stress and decrease in apoptosis [67,68]. A recent publication also suggests that cockroach allergen induces mitophagy in airway epithelia [69]. Additionally, the literature demonstrates that HDM induces reactive oxygen species (ROS) production and oxidative stress and damage of airway epithelia [70,71,72,73]. Our data indicate that DRP1 balances epithelial cell survival in response to HDM exposure, as shown by increased cleavage and activity of caspase 3 after *Drp1* deletion. This could suggest attenuated clearance of damaged mitochondria following *Drp1* deletion. DRP1-mediated mitochondrial fission facilitates clearance of damaged mitochondrial segments and reduces various stresses, thus suppressing cell death and protecting against barrier damage, though more mechanistic studies in the future could reveal the delicate balance of DRP1 action in allergic airway disease.

The human data on *DRP1* expression suggest that increases in *DRP1* may contribute to asthma severity. However, our work in mouse models indicated that *Drp1* expression is important for regulation of the inflammation and reactivity seen in airway disease, and that ablation and inhibition of DRP1 may not be beneficial in asthmatics. We therefore hypothesize that this increase in *DRP1* expression could be a byproduct of the increased stress and damage, which accrue in the airway epithelia of severe asthma patients. Furthermore, *DRP1* expression increases may be necessary to help to resolve the inflammatory responses triggered by HDM. More detailed studies are needed in mice and human samples to examine this phenomenon and correlate the data with clinical outcomes in asthmatics.

Collectively, our data indicate that DRP1-mediated mitochondrial fission is important for the regulation of airway epithelia pro-inflammatory response, as well as airway epithelial survival after exposure to HDM. This information and more mechanistic studies in the future will reveal the complex role of DRP1 in allergic airway disease.

## 4. Materials and Methods

### 4.1. Study Approval

All mouse studies were approved for use by the Institutional Animal Care and Use Committee of the University of Vermont under protocol number X9-016.

### 4.2. HDM and LPS

HDM (XPB70D3A2.5, Stallergenes Greer, Lenoir, NC, USA) was suspended in Phosphate Buffered Saline (PBS). HDM concentration was determined by protein concentration. LPS (LPS25, Sigma-Aldrich, St. Louis, MS, USA) was also diluted in PBS.

### 4.3. Human Bronchial Epithelial Cell Culture Experiments

HBEC-3KTs (CRL-4051, ATCC, Manassas, VA, USA) were plated in 2 mL DMEM/F12 (Gibco 11330057, Thermo Fisher Scientific, Waltham, MA, USA) with added growth factors at 4 × 10^6^ cells per dish in 35-mm dishes and allowed to adhere overnight. Cells were then exposed to either 50 μg HDM or PBS control, after a 2-h starvation period in DMEM/F12 without added growth factors. Cells were subsequently exposed to the same HDM concentration 48 h later, and supernatants and cell lysates were collected at corresponding time points. Cells used for immunofluorescence were plated on chamber slides (Nunc Lab-Tek II CC2 Chamber Slide System 154852, Thermo Fisher Scientific, Waltham, MA, USA) at 2.5 × 10^6^ cells per chamber and put through the same exposure protocol.

### 4.4. Primary Mouse Tracheal Epithelial Cell Culture Experiments

Primary MTECs were isolated and cultured, as previously described [74], from mice containing *LoxP* sites flanking exons 3 and 5 of *Drp1*. *Drp1^loxp/loxp^* mice were obtained from Dr. Hiroma Sesaki at Johns Hopkins University [13]. MTECs were plated in 35 mm dishes coated with 100 μg monomeric rat tail collagen (354236, Corning, Corning, NY, USA) at 7 × 10^6^ cells per dish and allowed to adhere overnight. Cells were infected with adenovirus expressing Cre recombinase (1700, Vector Biolabs, Malvern, PA, USA) at an MOI of 8 per cell to delete *Drp1* (adenovirus expressing an empty CMV promoter—1300, Vector Biolabs, Malvern, PA, USA—was used as a control). Cells were incubated for 3 to 5 days before being exposed to HDM in the same manner as described for the HBECs and supernatants and cell lysates were collected at corresponding time points. Cells used for immunofluorescence were plated on chamber slides coated with collagen at 4 × 10^6^ cells per chamber and subjected the same adenoviral and HDM protocols.

### 4.5. Transgenic Mice

Bitransgenic mice obtained from Dr. Whitsett at Cincinnati Children’s Hospital [75] were crossed with *Drp1^loxp/loxp^* mice. The mice contained two genetic inserts, i.e., rat club cell 10-kDa protein promoter linked to reverse tetracycline transactivator (*CC10-rtTA*) and a tetracycline operon linked with Cre recombinase (*TetOP-Cre*). The resulting mice (*CC10-rtTA+, TetOP-Cre+, Drp1^loxp/loxp^*) could deplete *Drp1* from club cells (*∆Epi-Drp1*) upon induction by doxycycline, introduced via mouse chow (6 g/kg; Purina Diet Tech, St Louis, MO, USA). Mice were given doxycycline-containing chow 10 days before HDM exposure and were maintained on doxycycline-containing chow for the duration of the experiment. Littermates missing one of the three genetic inserts (*CC10-rtTA+/TetOP-Cre+* or *CC10-rtTA+/Drp1^loxp/loxp^*) also given doxycycline-containing chow were used as controls (*Ctrl*).

### 4.6. AHR Assessment

Mice were anesthetized using sodium pentobarbital (90 mg/kg) via intraperitoneal injection and tracheotomized using 18-guage cannula. Mice were mechanically ventilated at a rate of 200 breaths/minute using a FlexiVent computer-controlled small-animal ventilator (SCIREQ, Montreal, QC, Canada). Newtonian resistance (Rn), tissue dampening (G), and tissue elastance (H) were measured in the mice after exposure to increasing concentrations of aerosolized methacholine. Results are shown as the average of three peak measurements with a COD value greater than 0.85.

### 4.7. Bronchoalveolar Lavage Fluid Processing

Bronchoalveolar lavage fluid (BALF) was collected by washing the airways with 1 mL of cold, sterile PBS. Cells were then isolated via centrifugation and total cell counts were determined by using a hemocytometer (3110, Hausser Scientific, Horsham, PA, USA). Cytospins were conducted and cells were stained using Hema3 stain reagents (Fisher Scientific, Waltham, MA, USA) to obtain differential cell counts. 300 cells minimum were counted to determine differential counts.

### 4.8. ELISAs

For cell culture experiments, supernatants were collected 24 h after the second HDM dose and used to assess secreted levels of IL8, IL6, CCL20, and G-CSF (DuoSet ELISA Kits, R&D Systems, Minneapolis, MN, USA) per the manufacturer’s instructions. For mouse experiments, right side lung lobes were flash frozen immediately after harvest and crushed to make lysates in buffer containing 137 mM Tris-HCL (pH 8.0) 130 mM NaCl, and 1% NP-40. Samples were normalized to total lung protein and used to assess expression levels of IL5, IL6, IL33, CXCL1, Eotaxin-1 (DuoSet ELISA Kits, R&D Systems, Minneapolis, MN, USA), IL4, IL13 (eBioscience Kits, Thermo Fisher Scientific, Waltham, MA, USA), and MUC5AC and MUC5B (Novus Biologicals, Littleton, CO, USA) per manufacturer’s instructions.

### 4.9. Western Blots

For cell culture experiments, cells were lysed in buffer containing 137 mM Tris-HCL (pH 8.0) 130 mM NaCl, and 1% NP-40. For mouse experiments lungs were flash frozen, pulverized and crushed and lysates were made in the same buffer. The desired soluble proteins were separated from insoluble proteins via ultracentrifugation. Following protein quantification, samples were resuspended in loading buffer with dithiothreitol (DTT) and resolved by SDS-PAGE. Proteins were transferred to PVDF and membranes were probed using standard immunoblotting protocol. Membranes were probed for phosphorylated DRP1 (pDRP1—3455S, Cell Signaling Technology, Danvers, MA, USA), total DRP1 (tDRP1—611113, BD, Franklin Lakes, NJ, USA), and β-actin (A5441, Sigma-Aldrich, St. Louis, MO, USA). Quantification of protein expression was determined by densitometry using ImageJ software (NIH, https://imagej.nih.gov/ij/, accessed on the 30 October 2018).

### 4.10. Immunofluorescence

For cell culture experiments, cells were fixed at corresponding time points following HDM exposure using freshly prepared 4% paraformaldehyde for 10 min and permeabilized for 30 min in 0.2% Tween-20 in PBS at room temperature. For mouse experiments, left lung lobes were formalin fixed overnight at 4 °C, mounted in paraffin, and sectioned at 5 μm thickness. Sections were fixed to glass slides and prepared for immunofluorescence by deparaffinization using xylene and rehydration through a series of ethanol washes. Lung antigen retrieval was achieved by submersing slides in sodium citrate buffer (pH 6.0) with 0.05% TWEEN-20 at 95 °C for 20 min. For both cells and lung sections, samples were blocked in 1% BSA in PBS for 1 h, followed by overnight incubation on primary antibody, diluted in PBS, at 4 °C. For cells, pDRP1 was used at a concentration of 1:200 and VDAC (Invitrogen PA1-954A) was used at 1:300. For lung sections, CC10 (Santa Cruz sc-390313) and cleaved caspase-3 (Cell Signaling Technology 9661) were used at a concentration of 1:300. Slides were then washed 3 × 5 min in PBS and subsequently incubated for 1 h at room temperature in fluorescent-conjugated secondary antibodies (Life Technologies) at half the concentration of the primary antibodies in 1% BSA in PBS. Samples were counterstained with DAPI at 1:4000 in 1% BSA in PBS for 10 min at room temperature and mounted using AquaPoly mounting medium (Polysciences 18606). Images were taken on a Nikon Laser Scanning Confocal Microscope (Nikon A1R-ER). Brightness and contrast were adjusted equally for corresponding images and images were analyzed for mean fluorescence intensity (MFI) and for mitochondrial form factor (FF = perimeter2/4π*area) using ImageJ software. For mitochondrial fission analysis, duplicate experiments were conducted with duplicate wells. Four to six images were taken per well, with each image containing at least 15 cells, allowing analysis of approximately 300 cells per group.

### 4.11. Analysis of mRNA Expression

*Muc5AC* (Forward–TCTACTGACTGCACCAACACAT; Reverse–GTGCAGTCCCCATTACTGT) and *Muc5B* (Forward–GCCTTGTCTCAGTCCCTCCTG; Reverse–TGACTGTCTCCGGTGAGTTCTA) were quantified in mouse by extracting RNA from flash frozen, pulverized left lung lobes using TRIzol (Invitrogen 15596018). RNA was purified using the RNeasy kit (Qiagen). 1 μg of RNA was reverse transcribed to cDNA (Promega) and SYBR Green Supermix (Bio-Rad) was used to quantify mRNA expression using RT-qPCR. For *Drp1* (Forward–AAGCCCTGAGCCAATCCATC; Reverse–CTCGATGTCCTTGGGCTGAT) quantification, lung epithelial cells were isolated from lungs of *Ctrl* or *∆Epi-Drp1* mice on doxycycline diet for 10 days using the GentleMACS lung dissociation kit (Miltenyi Biotech) followed by the EasySep mouse epithelial cell enrichment kit II (STEMCELL Technologies). Isolated epithelia were lysed in TRIzol and RNA was isolated and reverse transcribed in the same manner as whole lung lysates. *Drp1* expression in MTECs was also quantified by RT-qPCR following TRIzol lysis. Expression values were normalized to the geometric mean of *GAPDH* (Forward–AGGTCGGTGTGAACGGATTTG; Reverse–TGTAGACCATGTAGTTGAGGTCA), *PP1* (Forward–TTTTCATCTGCACTGCCAAG; Reverse–TCGAGTTGTCCACAGTCAGC), and *RP2* (Forward–TTGCCAGCAATTTCGTGTGA; Reverse–CCAGTTGAGCTCTCCTGACA) using the ∆∆CT method.

### 4.12. Mucus Metaplasia Quantification

Paraffin-embedded 5-μm tissue sections were mounted on slides, deparaffinized and rehydrated, and antigen retrieval was performed. PAS staining was conducted, and images were captured on a Leica VERSA8 whole slide imager. Mucus metaplasia was measured in the airways by measuring positive PAS-stained area using the Positive Pixel Count algorithm of Aperio ImageScope Software (Aperio Technologies, San Diego, CA, USA).

### 4.13. Caspase Assay

First, 25 μg of tissue lysates were diluted to 25 μL in dH2O and incubated with 25 μL Caspase-Glo 3/7 assay reagent (Promega) in an opaque plate in the dark at room temperature for 30 min. Total luminescence was measured using a Synergy HTX plate reader (Biotek) and values were recorded as relative activity.

### 4.14. Microarray Analysis

GEO2R (http://www.ncbi.nlm.nih.gov/geo/info/geo2r.html, accessed on the 9 July 2018) was used to compare differentially expressed genes between moderate and severe asthmatics, as classified by the American Thoracic Society, and nonasthmatic controls on GSE43696 [23,24]. GEO2R performs a base 2-log transformation.

### 4.15. Statistics

Outliers were determined using the ROUT method in GraphPad Prism 8 with a Q = 2%. The Shapiro-Wilk normality test was run. Microarray data were analyzed via nonparametric one-way ANOVA (Kruskal-Wallis test with Dunn’s multiple comparisons test). For the remainder of the data, normal data were analyzed by either two-tailed student’s t-test or two-way ANOVA, accordingly (T-test for comparisons of two groups, two-way ANOVA for comparisons of four groups). For two-way ANOVA analysis, Tukey’s post hoc test was used to adjust for multiple comparisons. If one or more of the groups did not pass the Shapiro-Wilk normality test, those data were analyzed by two-way ANOVA followed by the two-stage linear step-up procedure described by Benjamini, Krieger, and Yekutieli to control for false discovery rate. A *p*-value < 0.05 was considered significant. Data were averaged and expressed as the mean ± SEM.

## Data Availability

The human microarray dataset analyzed during the current study are available in the Gene Expression Omnibus (GEO) through NCBI, https://www.ncbi.nlm.nih.gov/geo/query/acc.cgi?acc=GSE43696, accessed on the 9 July 2018. All other data generated or analyzed are included in this publication or the Supplementary Information Files.

## Supplementary Materials

The following are available online at https://www.mdpi.com/article/10.3390/ijms222011125/s1.
