# Supplementary figures and images for: DRP1-Mediated Mitochondrial Fission Regulates Lung Epithelial Response to Allergen
Source: Int J Mol Sci. 2021 Oct 15;22(20):11125. doi: 10.3390/ijms222011125 (PMC8540036; doi:10.3390/ijms222011125)

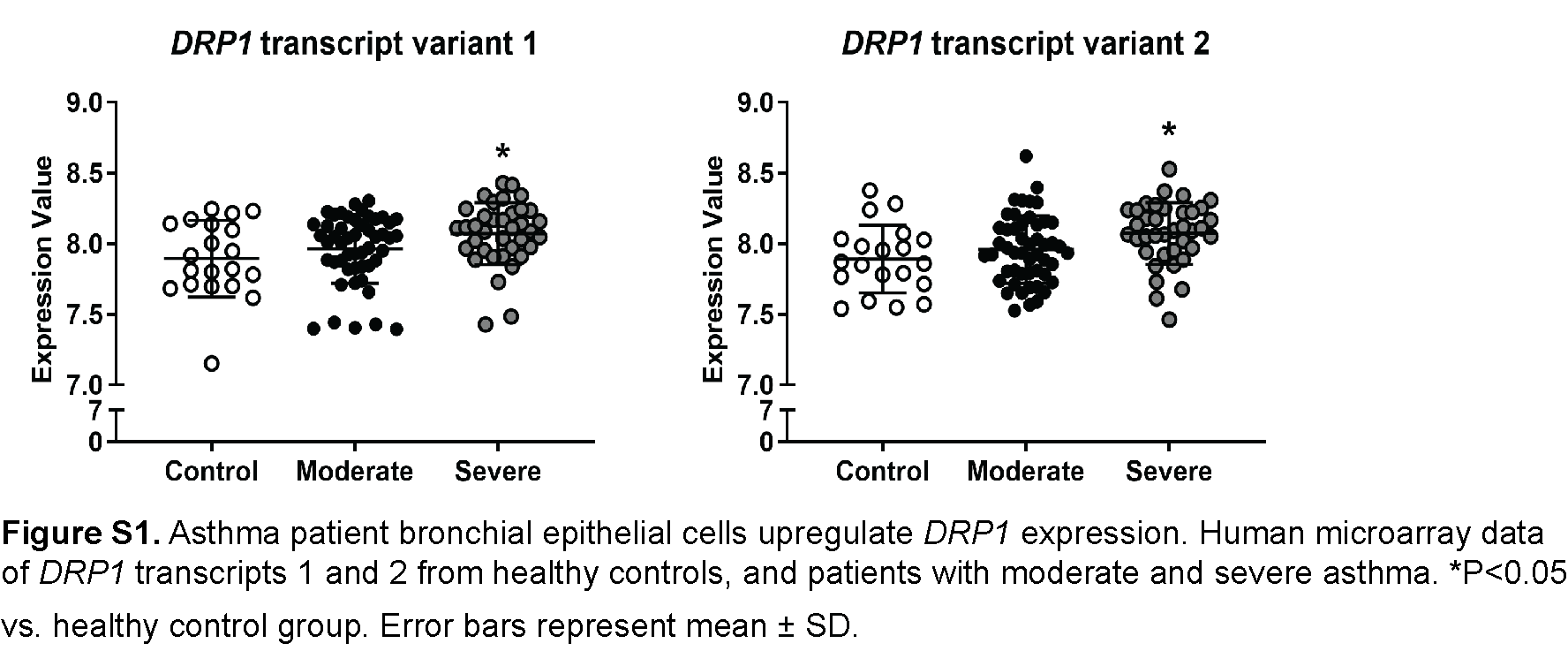

Supplement: Supplementary file 1 [file ijms-22-11125-s001.zip › Figure S1.tif]

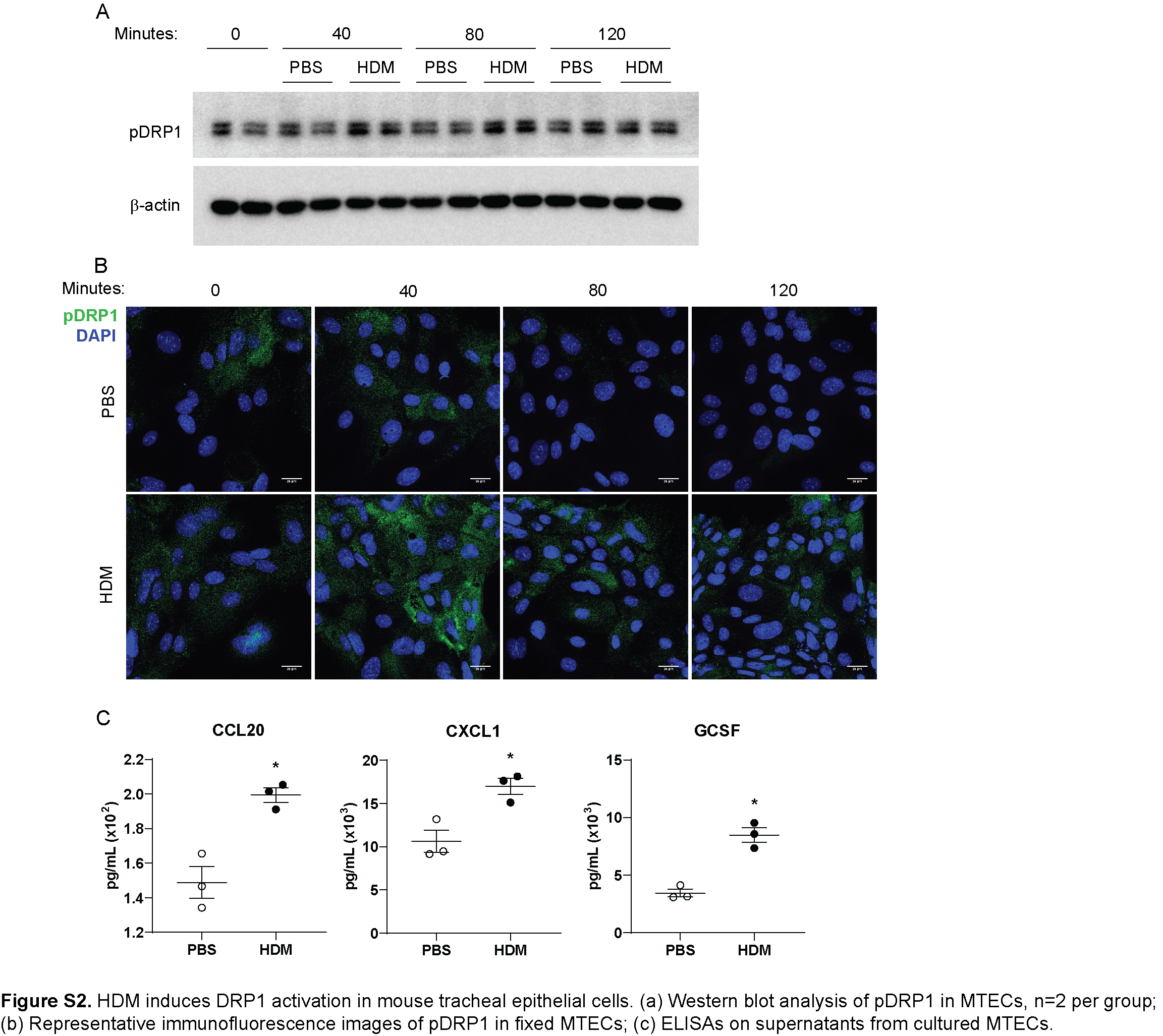

Supplement: Supplementary file 1 [file ijms-22-11125-s001.zip › Figure S2.tif]

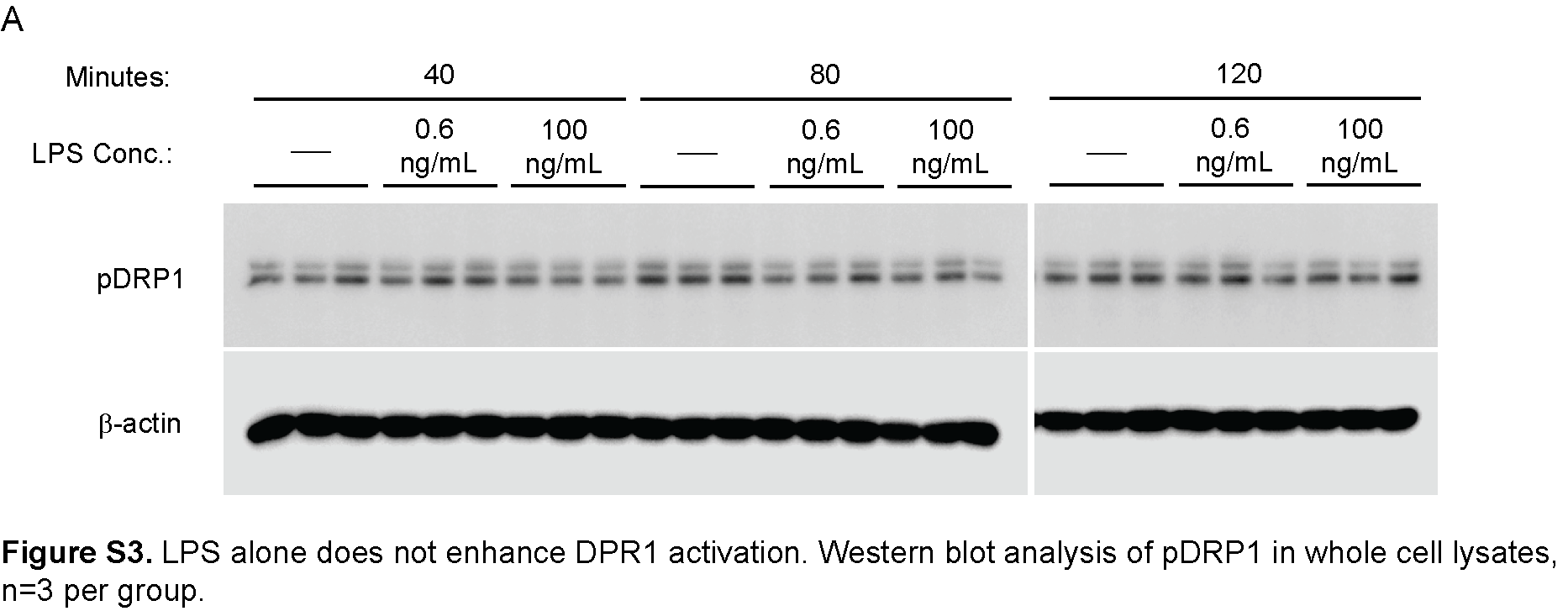

Supplement: Supplementary file 1 [file ijms-22-11125-s001.zip › Figure S3.tif]
